# Developing Social Entrepreneurship Orientation: The Impact of Internal Work Locus of Control and Bricolage

**DOI:** 10.3389/fpsyg.2022.877317

**Published:** 2022-05-26

**Authors:** Peng Xiabao, Emmanuel Mensah Horsey, Xiaofan Song, Rui Guo

**Affiliations:** School of Public Affairs, University of Science and Technology of China, Hefei, China

**Keywords:** internal work locus of control, entrepreneurial bricolage, social entrepreneurship orientation, core self evaluation theory, locus of control

## Abstract

Using core self-evaluation theory, the current study assesses the effect of internal work locus of control and bricolage on social entrepreneurship orientation. We adopted the cross-sectional survey design using a sampling frame to engage 400 top executives of social enterprises in mainland China. Three hundred and seventy-two of the executives replied, presenting a response rate of 93%. Results of structural equation modeling analysis show significant positive relationships between internal work locus of control, bricolage, and social entrepreneurship orientation. The positive mediating effect of bricolage on the relationship between internal work locus of control and social entrepreneurship orientation was also found to be true. Consequently, to foster social entrepreneurship orientation, top executives of social enterprises need to gather available resources for bricolage tasks. These findings contribute new knowledge to how internal work locus of control affects social entrepreneurship orientation through the bricolage activity of Chinese social enterprises. Through core self-evaluation theory, we demonstrate the effect of internal work locus of control as a preceding factor in the relationship between bricolage and social entrepreneurship orientation.

## Introduction

Over the past 15 years, there has been a fast-paced proliferation of research on social entrepreneurship based on its critical contribution to national and worldwide social, economic, cultural, and environmental wealth (Dato-on and Kalakay, 2016; Doherty, 2018; Bozhikin et al., 2019; van Lunenburg et al., 2020; Diaz Gonzalez and Dentchev, 2021). Specifically, social entrepreneurship has accounted for significant solutions either when viewed through its power of dealing with social problems in a traditional way, or *via* its powerful transformation of private-sector entrepreneurship (Bozhikin et al., 2019). This transforming power differentiates social entrepreneurship from traditional entrepreneurship in its primary mission of creating social value rather than generating private economic gains (Gupta et al., 2020). This impact reveals the mission of social enterprises. Social enterprises are setups that merge the pursuit of public social goods with market-aligned tools and techniques. They essentially function at the boundaries of the traditional philosophies of for-profits organizations (Mamabolo and Myres, 2020). Overall, social enterprises consider novel activities that intend to create producer surplus by reducing negative externalities and/or creating positive externalities *via* the integration of social as well as entrepreneurship constructs (Gupta et al., 2020). To these contributions, social enterprises have to generate earned income, engage stakeholders, create awareness about the social problems in their community, and attract government support. All of these are decisive factors in scaling up the social impact of a social enterprise (Bacq and Eddleston, 2018; Thorgren and Omorede, 2018). However, social enterprises’ successful function in these areas is being opposed by severe resources and capabilities constraints (Austin et al., 2012; Gupta et al., 2020).

Prior studies, in response, advanced our knowledge into the roles of several factors such as social entrepreneurs’ bricolage behavior (Kickul et al., 2018); social capital (Hidalgo et al., 2021); and non-governmental organizations (El Chaarani and Raimi, 2021) to address notable constraints in social entrepreneurship. Some key concerns from scholars pointed out that future research could assess the impact of market-related concepts such as entrepreneurial orientation. Scholars frequently stated that social purpose organizations, particularly social entrepreneurs should adopt entrepreneurial orientation to better counter constraints and accomplish social objectives (Pinheiro et al., 2021). Thus, systematic research is required in terms of defining the relationship between market (entrepreneurial) orientation and social performance (Kraus et al., 2017; Bhattarai et al., 2019). The central question about this focus is based on the well-established link between entrepreneurial orientation and performance in entrepreneurial studies, which has hitherto received little attention in social entrepreneurship (Halberstadt et al., 2021; Wales et al., 2021). Kraus et al. (2017) proposed the concept of social entrepreneurship orientation which is an integration of a social perspective into entrepreneurial orientation. Social entrepreneurship orientation is defined as the behavior that impacts social enterprises’ decision-making and practices in their discovery of new avenues to give unique solutions to societal issues (Gali et al., 2020). Despite the growing body of research on social entrepreneurship orientation, the literature has only explored its mediating and moderating impacts (Gali et al., 2020; Halberstadt et al., 2021; Pinheiro et al., 2021). This deprives us the insights into the evolution of social entrepreneurship orientation.

We investigate the roles of internal work locus of control and entrepreneurial bricolage as factors that influence social entrepreneurship orientation. Our proposal on these factors put forward the idea that internal work locus of control, a personality factor (Zigarmi et al., 2018; Robert and Vandenberghe, 2020) should explain what drives social entrepreneurs’ level of control in their activities and how they perceive the success of leveraging new approaches (bricolage) to cultivate new work behaviors (social entrepreneurship orientation). Spector (1988) defined internal work locus of control as a person’s belief that work outcomes such as task performance are determined by his or her actions. Internal work locus of control considers the degree of one’s personal view concerning the level of control in a specified work setting (Karkoulian et al., 2016; Turksoy and Tutuncu, 2021). Bricolage describes the creative recombination of existing resources to tackle resource shortages (An et al., 2018; Iqbal et al., 2021). Prior studies demonstrated that internal work locus of control positively influences job satisfaction (Wilski et al., 2015; Mulki and Lassk, 2019) as well as contributes to higher engagement and motivation in service delivery (Hu et al., 2016). Individuals’ proactiveness, innovativeness, and entrepreneurial abilities are also influenced by individuals’ internal work locus (Hsiao et al., 2016; Zhao and Wibowo, 2021). Although this significance has been given, the social entrepreneurship literature has fewer similar findings. Furthermore, there is substantial evidence that bricolage assists social entrepreneurs to deal with resource limitations (Crupi et al., 2021; Intindola and Ofstein, 2021) and leads to high-value items at low cost with inadequate resources *via* improvisation and experimental learning (Cai et al., 2019), yet little is known about how social entrepreneurs’ internal work locus of control, blends with bricolage to influence their social entrepreneurship orientation. Given this focus, the following research questions are addressed in this study: (1) For whom are internal work locus of control more beneficial? (2) How does internal work locus of control contribute to social entrepreneurship orientation?

We hypothesize two paths that can lead to the development of social entrepreneurship orientation in response to these questions. First, we propose a direct effect of internal work locus of control on bricolage. Second, a direct effect of bricolage on social entrepreneurship orientation. The former or first path follows the description and validation of an individual’s work locus (internality) as self-appraisal of tasks and the belief that one’s capabilities underlie one’s hard work (Karkoulian et al., 2016; Zigarmi et al., 2018). As a result, we anticipate that social entrepreneurs’ assessment of bricolage tasks and use will influence their possession of bricolage capabilities. The latter or second path is based on bricolage’s emphasis on concrete behaviors like innovativeness and resource constraint management. (Clough et al., 2019; Digan et al., 2019). Bricolage has been found to play several useful roles in social entrepreneurship, including assisting social entrepreneurs in overcoming resource constraints by making do with what they already have. Thus, we expect bricolage’s propensity to develop capabilities that describe social entrepreneurship orientation (social innovativeness, social proactiveness, social risk-taking, and socialness).

The role of bricolage in mediating the relationship between internal work locus of control and social entrepreneurship orientation is also investigated in this research. The assumption underlying this examination is that internal work locus of control may not directly influence social entrepreneurship orientation except through the learning approaches provided by bricolage. Senyard et al. (2014) pointed out that bricolage teaches how (process) to recombine available materials in novel ways to better meet demands. Other Scholars noted that bricolage is the means *via* which entrepreneurs handle problems or seize opportunities despite insufficient resources (Senyard et al., 2014; Bacq et al., 2015; Bojica et al., 2018; Janssen et al., 2018; Zollo et al., 2018). These emphases, taken together, should connect bricolage to social entrepreneurs’ social entrepreneurship orientation.

The current study makes two essential contributions. Theoretically, this study contributes to the social entrepreneurship literature by identifying internal work locus of control and bricolage as determinants of social entrepreneurship orientation. This insight lends support to the conceptualization of social entrepreneurship orientation (Kraus et al., 2017) and prior evidence of its impact on social enterprises’ performance (Gali et al., 2020). By demonstrating how social entrepreneurs’ internal work locus is related to bricolage, this study adds to the work locus of control literature (Wilski et al., 2015; Zigarmi et al., 2018). Similarly, we add to core self-evaluation theory’s highlights on the effect of personality variables in the workplace (Kacmar et al., 2009; Köppe and Schütz, 2019; Yoo and Lee, 2019). This shows that the bricolage task is determined by internality, which includes social entrepreneurs’ self-assessment and belief. The mediating role of bricolage enriches similar findings in the literature on its mediation impact (Yang, 2018). This emphasizes bricolage as the process that connects internal work locus to the evolution of social entrepreneurship orientation. Empirically, there has been no empirical research into the effects of internal work locus and bricolage on social entrepreneurs’ social entrepreneurship orientation in the Chinese context. Consequently, this research has implications for the conditions that underpin the role of internal work locus of control and bricolage in the development of social entrepreneurship orientation for Chinese social enterprises and similar occupational classes in other contexts.

The subsequent sections present: the theory and hypotheses, methodology, data analytical strategy, and results. The last sections report the theoretical and empirical implications, limitations of the study, and suggest future research directions.

## Literature Review

### Core Self-Evaluations Theory

In the current study, core self-determination theory offers a relevant theoretical foundation to explain the relationships between social entrepreneurs’ internal work locus of control, bricolage, and social entrepreneurship orientation. Core self-evaluation theory offers a unifying trait theory (Johnson et al., 2008) that has an influential effect on job characteristics (Judge, 1997). Core self-evaluation is a higher-order construct that comprises four lower-order traits: self-esteem, generalized self-efficacy, locus of control, and emotional stability (Aryee et al., 2017; Yoo and Lee, 2019). The theory asserts that individuals are motivated to behave in ways that are consistent with their self-image and that individuals with high self-esteem perform well to maintain their positive self-image (Zigarmi et al., 2018). Delving deeper into this theoretical view reveals that individuals with high core self-evaluation judge themselves in a consistently positive manner across settings, such as considering themselves as self-potent, self-worthy, anxiety-free, and in charge of their lives (Anand and Mishra, 2021).

Considerable evidence shows that core self-evaluation is positively associated with employees’ work attitudes and behaviors, such as job satisfaction, job performance, work-related motivation, and career success (Köppe and Schütz, 2019; Yoo and Lee, 2019; Anand and Mishra, 2021; Zhao and Wibowo, 2021). In our study, we consider the locus of control trait, rather than the overall core self-evaluation traits, because we aim to maximize the prediction of locus of control on task performance (bricolage). Scholars noted that each core self-evaluation trait relates to outcomes (Chen, 2012; Iles-Caven et al., 2020; Anand and Mishra, 2021). Precisely, core self-evaluation theory shows the extent to which individuals are convinced that they can control events themselves. This assumption, in the case of social entrepreneurs, can be explained as internality that stimulates their actions toward engaging in resource bricolage activities. In consequence, newly developed behaviors such as bricolage behavior would confirm the core self-evaluation proposal on traits that leads to human behaviors.

**FIGURE 1:**
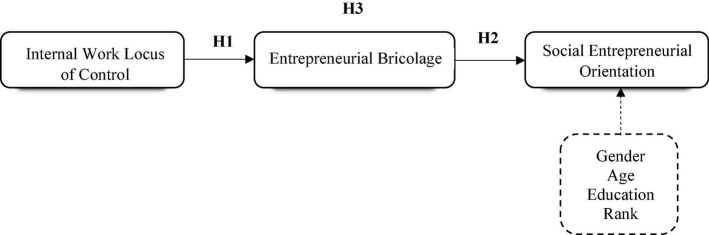
Conceptual model and hypotheses.

Given the fact that internal locus is associated with work attitudes, behaviors, and motivation, it offers a relevant basis to explain the interferences between social entrepreneurs’ internality and why they could consider bricolage. We show that the motivational characteristics of individuals’ internal locus of control influence them to find ways to cope with environmental factors and events. As control has been linked with active coping strategies and proactive behaviors, internality helps individuals to think and feel positive even in the occurrence of negative events (Aryee et al., 2017; Zhao and Wibowo, 2021). In this sense, social entrepreneurs will willingly engage in using whatever resources are at hand to construct social entrepreneurship orientation. Based on the core self-evaluation theory such desired behavioral outcomes are linked to an individual’s traits. The conceptualized relationships of this study are shown in Figure 1.

### Locus of Control and Work Locus of Control

Rotter (1966) coined the term locus of control to describe the degree to which individuals assign the causes of events or the outcomes of success and failure to their activities or other forces. In addition, locus of control covers the extent to which an individual considers rewards either contingent upon one’s behavior (internal locus of control) or controlled by forces outside of the individual (external locus of control) (Rotter, 1966). This implies that an individual may prefer or exhibit either of the two types of control, for example, in the case of internal locus of control, an individual will direct the causes or consequences of events toward self, whereas, in the case of an external locus, the individual will direct the outcomes such as coincidental or by chance from the perspective of forces outside him or herself (Wilski et al., 2015; Zigarmi et al., 2018). Thus, locus of control relates to the degree to which results appear to be dependent on a person’s behavior. Locus of control has a well-established history throughout the literature on personality theory (Ryon and Gleason, 2014; Zigarmi et al., 2018). Scholars, for instance, Iles-Caven et al. (2020) noted that internality is related to more positive outcomes such as religious behavior and belief than externality.

A further conceptualization of locus of control was tailored to an individual’s work context (Spector, 1988). The term work locus of control generally describes the extent of one’s personal view concerning the level of control in a specified work environment. Work locus of control also comprises two indicators: internal and external work locus of control. Individuals who have an internal work locus of control believe that their actions impact outcomes such as task performance, and enhance leader-member exchange relationships (Robert and Vandenberghe, 2020). Spector (1988) indicated that individuals with high internal work locus of control demonstrate superior leadership abilities, motivation, contentment, and performance. External work locus of control describes an individual’s feeling that several externalities are responsible for the individual’s work outcomes. Between these two, some scholars claimed that internal work locus of control has more impact than external work locus of control (Wilski et al., 2015; Mulki and Lassk, 2019). Some authors have established the importance of work locus of control as a construct by empirically examining the difference between general locus of control and work locus of control (Zigarmi et al., 2018). They indicated that the general concept of locus of control has significant relationships with general criteria such as affective commitment and life satisfaction, whereas work locus of control had comparatively stronger relationships with job satisfaction, affective commitment, and burnout, above and beyond the variance in these work outcomes explained by general locus of control (Zigarmi et al., 2018). We leverage internal work locus of control as a personal factor (Spector, 1988). Drawing on the significant impact social entrepreneurs create under unfavorable conditions (Austin et al., 2012), this study seeks to analyze the role of their internal work locus on task performance (bricolage) and the evolution of countering behavior (social entrepreneurship orientation).

### Social Entrepreneurship Orientation

Social entrepreneurship orientation refers to the behavior that influences social entrepreneurs’ decision-making and practices as they explore new paths to offer exceptional solutions to societal challenges (Gali et al., 2020). Following the significant contribution of entrepreneurial orientation to understanding the performance of commercial enterprises, social entrepreneurship orientation was developed based on dimensions of entrepreneurial orientation with the addition of a social perspective (Kraus et al., 2017).

Exploring the link and applicability of entrepreneurship theories and constructs to the context of social entrepreneurship has influenced the call to integrate entrepreneurial orientation (Dey and Steyaert, 2012; Choi and Majumdar, 2014). Nonetheless, there have been few scholarly attempts to examine the impact of social entrepreneurship orientation on social enterprise performance (Gali et al., 2020; Halberstadt et al., 2021; Pinheiro et al., 2021). The term “entrepreneurial orientation” refers to an enterprise’s entrepreneurial processes, practices, and decision-making activities that influence new market entry (Lumpkin and Dess, 1996). Entrepreneurial orientation has become a key concept in entrepreneurship research and has been discussed extensively (Covin and Wales, 2012). Thus, similar contributions sought after in social entrepreneurship stems from the field’s duality, which is its integration of entrepreneurial activity with social goals (Kraus et al., 2014).

### Bricolage

The term “bricolage” describes the use of existing resources and repertoires to complete any kind of tasks that emerge (Visscher et al., 2018; Iqbal et al., 2021; Simba et al., 2021). In other words, bricolage refers to the innovative recombination of preexisting resources to address the demands of one’s entrepreneurial activity. In this regard, an individual who utilizes bricolage or engages in resource recombination activities is known as a bricoleur (Visscher et al., 2018). Similarly, Weick (1993) defined a “bricoleur” as an individual who expresses creativity in a chaotic environment by using whatever materials available to create a novel combination. Baker et al. (2003) described bricolage as “making do” by applying existing resources to new problems or opportunities. “Making do” is a concept that seeks to understand the “rules” of individuals engaged in bricolage activity or “game” as constantly making do with “whatever resources are at hand.” (Visscher et al., 2018).

In the literature, bricolage takes two forms: internal and external bricolage. Scholars noted that both types are vital for an enterprise’s long-term success (Padilla-Melendez et al., 2020; Liu et al., 2021). The first classification refers to an enterprise’s or entrepreneur’s internal resource pool, which includes past knowledge, experience, education, and credentials (Padilla-Melendez et al., 2020). Precisely, this classification emphasizes that internal resources must be usable, manipulated, improvised, and deployed in an enterprise’s operational and management processes. Second, external bricolage refers to activities that increase the pool of potential resources available to entrepreneurs in their external networks, such as inter-organizational physical assets, functional assets, and social relationships (Perkmann and Spicer, 2014). According to scholars, network bricolage enables entrepreneurs who face institutional constraints to reconfigure available resources to overcome these constraints (Desa, 2012; Perkmann and Spicer, 2014). A large body of research agrees on the critical relevance of bricolage in chasing possibilities in a resource-constrained context (An et al., 2018; Iqbal et al., 2021). Prior studies found that bricolage implementation in resource-constrained enterprises results in frugal innovation (Ernst et al., 2015). The knowledge that emanates from bricolage enables enterprises to break resource inertia and stimulate creative inventions. Moreover, enterprises with stronger bricolage are more likely to develop low-cost, value-added goods and services for customers through improvisation and experimental learning (Cai et al., 2019).

Other operationalization of bricolage includes material and labor bricolage (Desa, 2012; Perkmann and Spicer, 2014; Castellani and Roca, 2021). Material bricolage refers to materials that have been neglected, discarded, worn, or committed to a specific use but can be used through creative recombination (Desa, 2012). By contrast, labor bricolage refers to human resources such as employees, customers, suppliers, and other human capital that are used as input to an enterprise’s projects (Desa, 2012; Castellani and Roca, 2021). These types of bricolage work together to help social enterprises make the necessary changes or adjustments to improve their performance (Intindola and Ofstein, 2021).

### Hypotheses Development

#### Internal Work Locus of Control and Bricolage

Prior studies documented the positive effects of internal work locus of control on studied outcome variables such as affective commitment, well-being, and job performance (Wilski et al., 2015; Mulki and Lassk, 2019). Similarly, prior findings indicated that individuals’ internal work locus of control is related to higher participation and motivation in service delivery (Hu et al., 2016), better job satisfaction (Tillman et al., 2010), and good ethical climate perceptions (Domino et al., 2015). Scholars also found that individuals with high internality are proactive, innovative, and possess higher entrepreneurial skills (Hsiao et al., 2016).

According to the literature on social entrepreneurship, “bricoleurs” have key entrepreneurial skills that speed up the development of new enterprises and enable them to overcome resource limitations (Linna, 2013; Senyard et al., 2014; Bacq et al., 2015; Servantie and Rispal, 2018). These capabilities are a result of their engagement in bricolage resource orchestrations. Furthermore, “bricoleurs”’ capabilities provide enterprises with optional, reliable alternatives, (Bojica et al., 2018), and relieve them from single reliance on government, stakeholders, and donor support (Pinheiro et al., 2021).

Along with this evidence, this study expects internal work locus of control to help explain social entrepreneurs’ bricolage task participation and related capabilities, which include: (1) being able to effectively use resources at hand through innovative recombination; and (2) improvise resources when actual resources are scarce. Reflecting on social entrepreneurs’ severe resource constraints (Austin et al., 2012; Gupta et al., 2020), the internality of social entrepreneurs should be a strategic approach to explain how such constraints can be addressed *via* tasks and outcomes. Consistent with extant findings, individuals may lessen uncertainty (perceived constraints) by exercising internal work locus of control rather than waiting for support (Peltokorpi et al., 2022). To conclude, scholars noted that individuals’ attribution of successful work outcomes being a result of their behavior or action drives them to learn and succeed with tasks (e.g., bricolage) as well as overcome negative experiences (Zigarmi et al., 2018) such as severe resource constraints. Along with these shreds of arguments, this study hypothesizes that:

***Hypothesis 1:** Internal work locus of control is positively related to bricolage.*

#### Bricolage and Social Entrepreneurship Orientation

Prior research demonstrated the usefulness of resource bricolage in social entrepreneurship as a means for social entrepreneurs to scale and tackle extensive issues such as world hunger or generational poverty by combining resources they come across (Intindola and Ofstein, 2021). Also, the integration of bricolage in social entrepreneurship has aided in the identification of ground-breaking and useful solutions using only available and occasionally worthless resources to bring about positive social change in societies (Crupi et al., 2021; Intindola and Ofstein, 2021). The creative adoption and manipulation of resources comprised human and social capital, financial, and material to create new opportunities (An et al., 2018; Nor-Aishah et al., 2020).

Drawing on this study’s Hypothesis 1, the authors further argue that social entrepreneurs with an internal work locus of control should not only be better able to deal with bricolage resource reconfiguration, but they should also demonstrate social entrepreneurial orientation capabilities such as social innovativeness, social proactiveness, social risk-taking, and socialness. In this sense, the link between bricolage and social entrepreneurial orientation can be explained by bricolage’s proclivity for causing the development of this orientation through experimenting (learning) with readily available resources. This idea is based on previous discoveries that bricolage refers to an individual’s (bricoleur) resource management behavior (Sharmelly and Ray, 2018; Nor-Aishah et al., 2020). In the process of the bricolage approach, individuals develop and employ essential skills required to make simple, low-cost items and services (Kickul et al., 2018; Sharmelly and Ray, 2018). Thus, the intensity of bricolage is proportional to an individual’s subjective knowledge of resource utilization, with important implications for product development, business endeavors, and strategic renewal (An et al., 2018).

This study expects that the consequent innovativeness of the bricoleur (Senyard et al., 2014) describes social innovativeness, which refers to the frequent strategic renewals or idea generation (An et al., 2018). Scholars stressed that bricolage encourages enterprises to discover new chances and enter markets before their counterparts/competitors do (Salunke et al., 2013; Yu et al., 2020). This evidence backs up our idea that bricolage would impact social proactiveness. Due to bricolage’s tendency for creating more with less (e.g., recycle waste), there is a likelihood of taking risks more readily (Sharmelly and Ray, 2018). Bricolage should also influence socialness, which stresses achieving social goals through partnerships. Along with these shreds of arguments, the following hypothesis was developed.

***Hypothesis 2:** Bricolage positively influences social entrepreneurship orientation.*

#### The Mediating Role of Bricolage

Although research on internal work locus of control has shown that it is linked to behavioral outcomes such as affective commitment and job performance (Wilski et al., 2015; Mulki and Lassk, 2019), this study argues that it may not have a direct impact on social entrepreneurship orientation. The different components that make up social entrepreneurship orientation lead to this argument. We show that developing this orientation is critical to an individual’s participation in a learning process, for instance, extant approval of bricoleurs’ resource recombination processes (Fisher, 2012; Iqbal et al., 2021; Simba et al., 2021). Specifically, we identify bricolage as a means through which internal work of control contributes to developing social entrepreneurship orientation. Ample empirical findings validate this assumption. First, bricolage, as a resource orchestration technique, considerably improves a new venture’s strategic flexibility and growth capabilities (Nor-Aishah et al., 2020). Second, bricolage focuses on taking action and actively participating in opportunities or challenges rather than debating whether or not feasible solutions can be achieved with current resources (Senyard et al., 2014; Janssen et al., 2018; Zollo et al., 2018). Last, scholars asserted that the use of available resources (process) for new purposes produces positive outcomes (Desa and Basu, 2013; Clough et al., 2019). For these reasons, this research claims that bricolage is critical in connecting the benefits of internal work locus of control to social entrepreneurship orientation. As a result, it is appropriate to hypothesize that:

***Hypothesis 3:** Bricolage positively mediates the relationship between internal work locus of control and social entrepreneurship orientation such as it serves as means of developing this orientation.*

## Materials and Methods

### Data Source

The current study adopted the cross-sectional survey design which is consistent with recent studies conducted in China, for instance, Li et al. (2021). Our sample comprised top executives of social enterprises located in mainland China. Due to this study’s assumption regarding internal work locus of control which is a personality factor (Ryon and Gleason, 2014), the top executives were suitable samples since they could report on how their internality influences their task (bricolage) and the evolution of capabilities (social entrepreneurship orientation). To avoid ambiguity, this study’s questionnaire was prepared in such a way that each portion proceeded with detailed instructions. This method was utilized to control response bias because this study used the cross-sectional survey design to gather data (Podsakoff et al., 2003). The questionnaire was translated from its original English version into Chinese simplified language, checked for clarity, and then back-translated into English to ascertain conceptual similarity before dissemination (Kriauciunas et al., 2011).

To determine our study participants, experts inside and outside the authors’ School of Public Affairs at the University of Science and Technology of China were consulted. We developed a sampling frame from a social/non-profit enterprise database in mainland China. The following sampling criteria were used to select the appropriate enterprises: (1) enterprises with embedded social and economic purpose; (2) have been in operation at least three years and beyond; (3) are independently owned; (4) have made a significant social and economic impact. From these selection criteria, 400 out of 937 social enterprises qualified for our study. The questionnaire was disseminated to top executives through email and WeChat. To encourage collaboration and prompt responses, gentle reminders were delivered regularly. The survey was conducted over eight months, from March to October 2021.

This study uses the Chinese context to validate the effect of internal work locus, and bricolage on social entrepreneurship orientation since China has been experiencing fast growth in social entrepreneurship (Qin et al., 2017). Social entrepreneurship emerged in China in 2004, and the idea of entrepreneurial activity has been adopted by most social-related institutions to actualize their missions (Wang et al., 2016). Against this background, it would be beneficial to Chinese social enterprises to develop social entrepreneurship orientation to succeed with their entrepreneurial activities and proliferate social value.

In total, 400 copies of survey questionnaires were sent out and 372 were retrieved successfully. The recovery rate of effective questionnaires was 93%. Among the final sample, 182 (48.9%) were female and 190 (51.1%) were male. 155 (41.7%) respondents were between the age of 26 to 35. The vast majority were master’s degree holders representing 76.9% of the total sample. Most respondents were executive directors (65.1%) of their enterprises. Table 1 summarizes the demographic statistics of our sample.

**TABLE 1:** Sample demographics.

Variables	Category	Frequency	Percent
Gender	Male	190	51.1
	Female	182	48.9
Age	25 and below	38	10.2
	26–35	155	41.7
	36–45	111	29.8
	46 and above	68	18.3
Rank	Bachelor’s	50	13.4
	Master’s	286	76.9
	Doctorate	36	9.7
Education	Executive Director	242	65.1
	Director	92	24.7
	Associate Director	38	10.2
	Total	372	100

### Measurements

The current study adopted a well-validated scale measurement from top-tier journal publications to measure the three constructs comprising internal work locus of control, bricolage, and social entrepreneurship orientation. To be precise, eleven items were adopted from the works of Kraus et al. (2017) and Gali et al. (2020) to measure social entrepreneurship orientation. Four items from Spector (1988) measured internal work locus of control. These four items were suitable for this study’s argument of the effect of individuals’ internality on bricolage. Bricolage was measured using nine items adopted from the work of Baker and Nelson (2005). Each of these items was rated on a 7-point Likert scale ranging from 1 = strongly disagree to 7 = strongly agree.

### Control Variables

Gender, age, education, and rank were controlled following prior studies’ endorsement of their possible effects on individuals’ attitudes and behaviors. Specifically, gender, age, and education were found to influence entrepreneurial behavior and success (Barrick et al., 1994; Bosma and Levie, 2010). Rank or position was controlled based on its effect on individuals’ orientation (Burt, 1997).

### Data Analysis

We adopted the structural equation modeling technique using the Analysis of Moment of Structures (AMOS) software version 24.0 (Fan et al., 2022). Before testing the hypothesized relationships of this study, calculations were made to check the reliability, convergent, and discriminant validity among the multi-item constructs as well as common method variance and variance inflation factor analysis to ascertain the levels of response bias and multicollinearity issues. Subsequently, Pearson’s correlation analyses were carried out to measure the strength of the linear relationship between constructs. The correlation analysis laid a foundation for meaningful hypothesis testing and results. The hypotheses were also estimated using structural equation modeling.

## Results

### Common Method Variance

Due to this study’s use of cross-sectional survey data, particularly from a single source and common scale properties, we employed Harman (1967) one-factor test to check for the tendency of common method bias. Along with the condition of no factor rotation, the cumulative percentage of 41.39% obtained was below the recommended threshold of < 50% (Podsakoff and Organ, 1986). This indicated that the common method variance is not an issue in this study. In addition, a full collinearity test was conducted following the recommendation of Kock and Lynn (2012). This estimation was to decipher whether two or more variables are collinear. As shown in Table 2, the variance inflation factor (VIF) values generated were lower than the cutoff point of 3.3 (Petter et al., 2007).

**TABLE 2:** Means, standard deviations, and correlations of variables.

Variables	1	2	3	4	5	6	7
Gender	1						
Age	0.094	1					
Rank	–0.002	0.216**	1				
Education	–0.002	0.003	–0.039	1			
IWLC	0.141**	0.248**	0.109*	0.024	**(0.788)**		
EB	0.036	0.037	0.055	–0.015	0.194**	**(0.810)**	
SEO	0.013	0.133*	0.075	–0.01	0.356**	0.254**	**(0.857)**
*M*	1.49	2.56	1.96	1.45	3.76	4.05	4.39
*SD*	0.501	0.905	0.480	0.673	0.741	0.888	1.043
*VIF*	1.033	1.122	1.055	1.002	1.051	1.150	

*n = 372.*

*Numbers in parentheses on the diagonal are reliabilities of these variables.*

*Indicates p < 0.05, **indicates p < 0.01, ***indicates p < 0.001.

*IWLC, internal work locus of control; EB, entrepreneurial bricolage; SEO, social entrepreneurship orientation. Square Root of AVE Values are bolded and bracketed.*

### Confirmatory Factor Analysis

We performed confirmatory factor analysis using AMOS 24.0 software. The three-factor model fit indexes (χ2 = 875.202, df = 249, TLI = 0.914, CFI = 0.922, RMSEA = 0.082) were better compared to the other models reported Table 3. This indicates that all variables in the conceptual model had good discriminant validity. Also, the standardized factor loadings of all items in the three-factor model were above 0.7. This provided additional support for the convergent validity of the three variables.

**TABLE 3:** Results of confirmatory factor analysis.

Model	Model combination	*x* ^2^	df	*x*^2^/df	RMSEA	NFI	TLI	CFI
One-factor model	IWL + EB + SEO	3822.629	252	15.169	0.195	0.541	0.515	0.557
Two-factor model	IWL;EB + SEO	3236.975	251	12.896	0.179	0.612	0.593	0.630
Three-factor model	IWL;EB;SEO	875.202	249	3.515	0.082	0.895	0.914	0.922

*IWL, internal work locus; EB, entrepreneurial bricolage; SEO, social entrepreneurship orientation.*

Furthermore, we calculated the extracted mean variance values of three variables. Table 3 illustrates the arithmetic square root of the extracted mean-variance values. All values were above 0.5 which indicates that the three variables in this study had satisfactory convergence validity. In the same vein, the arithmetic square root of the extracted mean-variance values of the three variables was all above the correlation coefficient between these variables and other variables. This confirmed good discriminant validity between the core constructs in this study.

### Correlation Analysis

Table 2 reports the descriptive statistics and correlation matrix with Pearson Correlation Coefficients (*r*) of all the variables. Pearson’s approach was used to measure the strength of the linear relationships. At a significant level of 0.05, social entrepreneurship orientation positively correlated with internal work locus of control and bricolage. Table 4 provides details of correlations between variables.

**TABLE 4:** Path analysis of structural model.

Hypothesis	Path	Coefficient	*t*-value	*P*-value	Interpretation
H1	IWLC→EB	0.226***	3.816	***	Supported
H2	EB→SEO	0.350***	4.807	***	Supported
	Control variables	–0.006	–0.059	0.953	
	Gender→SEO	–0.006	–0.059	0.953	Insignificant
	Age→SEO	0.130*	2.307	0.021	Significant
	Rank→SEO	0.069	0.656	0.512	Insignificant
	Education→SEO	–0.012	–0.169	0.866	Insignificant
	Construct	R2			
	EB	0.050			
	SEO	0.092			

IWLC, internal work locus of control; EB, entrepreneurial bricolage; SEO, social entrepreneurship orientation. *Indicates p < 0.05, **indicates p < 0.01, ***indicates p < 0.001.

### Hypothesis Testing

Consistent with prior analytical techniques, the structural equation modeling was used to test the hypotheses in this study (Arkorful and Lugu, 2021; Fan et al., 2022). All indexes met acceptable standards (χ^2^/df = 2.386, IFI = 0.967, NFI = 0.930, TLI = 0.961, CFI = 0.967, GFI = 0.901, RMSEA = 0.048). The standardized fitting results are shown in Table 5.

**TABLE 5:** The goodness of fit indexes measurement.

Categories	Indexes	Threshold	Results
Absolute fit	RMSEA	<0.08	0.048
	GFI	>0.90	0.901
	CFI	>0.90	0.967
Incremental fit	TLI	>0.90	0.961
	NFI	>0.90	0.930
	IFI	>0.90	0.967
Parsimonious fit	Chisp/df	<5	2.386

As reported in Table 4, Hypothesis 1 which states that internal work locus of control is positively related to bricolage was tested. We found a significant positive correlation for this relationship (β = 0.479, *p* < 0.01). This means Hypothesis 1 was valid. Also, Hypothesis 2 which states that bricolage positively influences social entrepreneurship orientation was found to be significantly positive (β = 0.479, *p* < 0.01.) Thus Hypotheses 2 was supported.

**FIGURE 2:**
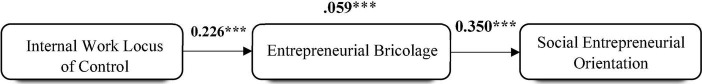
Hypotheses testing estimates. ***indicates *p* < 0.001.

We further estimated the mediating effect of bricolage using the structural equation modeling through the bootstrapping test method (Taylor et al., 2008). The results of mediation analysis (Table 6), concerning Hypothesis 3, which states that bricolage positively mediates the relationship between internal work locus of control and social entrepreneurship orientation such as it serves as means of developing this orientation was supported with a significant positive value of β = 0.059, [95% CI: 0.017–0.118]. Figure 2 summarizes the Hypotheses testing estimates of this study.

**TABLE 6:** Bootstrap test for mediating effect.

Hypothesis	Paths	Coefficient	Bias-corrected 95%CI		Results
			
		(β)	Lower	Upper	
H3	IWLC→EB→SEO	0.059	0.017	0.118	Supported

*IWLC, internal work locus of control; EB, entrepreneurial bricolage; SEO, social entrepreneurial orientation.*

## Discussion

The current study hypothesized and tested how social entrepreneurs’ internal work locus of control relates to bricolage, as well as bricolage’s impact on Chinese social entrepreneurs’ social entrepreneurship orientation. This research focus was triggered by prior emphases on social entrepreneurship orientation (Kraus et al., 2017; Bhattarai et al., 2019) and the fewer insights in the social entrepreneurship literature about its determinants. Addressing this gap was anchored on the research questions: (1) For whom are internal work locus of control more beneficial? (2) How does internal work locus of control contribute to social entrepreneurship orientation?

The current study’s findings revealed that internal work locus of control positively impacts bricolage as well as bricolage’s positive influence on social entrepreneurship orientation. Also, bricolage positively mediated the relationship between internal work locus of control and social entrepreneurial orientation. These outcomes are interpreted as follows: First, the positive linkage between internal work locus of control and bricolage infers that social entrepreneurs’ perception of their hard work and its consequences promote bricolage task performance. In this sense, they willingly engage in resource reconfiguration using available resources. Along with this conclusion, the current research adds to the body of knowledge on work locus of control (Ryon and Gleason, 2014; Wilski et al., 2015; Zigarmi et al., 2018) by demonstrating how social entrepreneurs’ internality is linked to bricolage activities. Instead of the dominant application of internal work locus of control to psychology and entrepreneurship research (Wilski et al., 2015; Zigarmi et al., 2018), this insight shifts the conservation to social entrepreneurship (Kraus et al., 2017).

Second, the positive impact of bricolage on social entrepreneurship orientation implies that capabilities such as social innovativeness and social proactiveness are developed during social entrepreneurs’ resource reconfiguration process. In this sense, each social market demand sparks fresh ideas for repurposing existing resources which facilitate the effect of bricolage on social entrepreneurship orientation. This finding enriches prior research documentation on the role of bricolage in developing resource constraints countering behavior (Senyard et al., 2014; Bacq et al., 2015; Bojica et al., 2018).

Third, the positive mediating role of bricolage on the link between internal work locus of control and social entrepreneurship orientation infers that social entrepreneurs’ internality connects to social entrepreneurship orientation through bricolage. In this vein, bricolage becomes the learning platform where social entrepreneurs learn how to recombine and improvise preexisting resources. Consequently, this makes them innovative, proactive, better able to take risks and enter partnerships to accomplish their social objectives. Following the mixture of arguments regarding the effect of bricolage (positive, neutral, or negative effects) (Baker and Nelson, 2005), this study adds to the literature on bricolage’s positive mediating role (Yang, 2018) in the internal work locus of control and social entrepreneurship orientation relationship.

### Theoretical Implications

The current study contributes to core self-evaluation theory (Judge, 1997; Johnson et al., 2008) in several ways. First, our study emphasizes internal work of control as an important determinant of bricolage. Thus, we extend the limited research on the understanding of the effect of core self-evaluation traits on key workplace factors (Kacmar et al., 2009; Roberts and Zigarmi, 2014; Ozcelik and Barsade, 2018; Yoo and Lee, 2019).

Second, no previous research to the best of the authors’ knowledge has studied the effects of internal work locus of control on social entrepreneurs’ bricolage and social entrepreneurship orientation. In general, prior research showed that individuals’ core self-evaluation affects individuals’ work attitudes and behaviors, such as job performance, work-related motivation, leader-member relationships, and entrepreneurial abilities (Chen, 2012; Iles-Caven et al., 2020; Anand and Mishra, 2021; Zhao and Wibowo, 2021) but fewer insights exist in social entrepreneurship.

Third, our examination of the mediating effect of bricolage between internal work locus of control and social entrepreneurship orientation advances the core self-evaluation theory by providing a new understanding of the mechanism through which internal work locus of control impacts social entrepreneurship orientation. Although existing research found that individuals’ internality impacts their active coping strategies, proactive behaviors, and positive feelings (Aryee et al., 2017; Köppe and Schütz, 2019; Zhao and Wibowo, 2021), little is known about the mechanisms that serve as the mediation between internal work locus of control and these behaviors.

### Empirical Implications

Empirical research on the roles of internal work locus of control and bricolage in social entrepreneurship is notably limited in mainland China. To be more specific, no study has examined how these factors influence the social entrepreneurship orientation of Chinese social entrepreneurs. In addition, the concept of social entrepreneurship in China is at its early stage compared to other contexts (Wang et al., 2016), as a result, our empirical findings provide Chinese social entrepreneurs with a reliable strategy to increase the outcomes of social entrepreneurs internal work locus *via* a resource experimental learning to construct capability that assures and sustains social and economic performance.

Considering the current study’s findings, the practical implications come in two folds. First, the findings suggest that social entrepreneurs’ internality influences their adherence to bricolage which leads to the possession of capabilities such as social innovativeness. Therefore, top executives of social enterprises should foster bricolage by gathering internal and external resources. Due to the severity of social entrepreneurs’ resource constraints (Austin et al., 2012) such resource-based initiatives would foster bricolage activity.

Second, top executives of social enterprises should leverage bricolage as a long-term learning approach since the resource reconfiguration process and development of capabilities could be realized after long periods. This is subjective to the internality of the bricoleur or individual engaged in bricolage resource reconfigurations. Therefore, top executives should have an organizational learning schedule that permits regular learning on bricolage. Some of these learning initiatives can focus on increasing knowledge of and access to education, providing experience-based learning opportunities, and connecting to external networks to augment the effect of bricolage on social entrepreneurship orientation.

### Limitations and Suggestions for Future Research

Due to the nature of the sampling context, the current study has limitations that create avenues for future studies. Although we utilized a suitable sample and self-reported measure, our sample validated the propositions of this study. However, the lack of time series/panel data paves the way for future investigations. This would contribute to generalizing the results of the current study. This study confined its investigation to social entrepreneurs in the Chinese context. Despite this limitation, this study’s findings are robust considering the reliability and validity tests conducted. Future studies could consider diverse or comparative empirical analyses to decipher whether these results are consistent across countries. Though this study’s results may be applicable in other contexts, such scope would help understand whether contextual differences such as culture underlies the causality of these studied determinants. Besides, this study’s conceptual framework is novel, as a result, it paves the way for diverse investigations. Taking this viewpoint into consideration, future studies could investigate whether culture influences the relationship between internal work locus of control and bricolage. Last, future research could also analyze other relevant variables such as political and business ties as conduits for resource mobilization among others that may probably mediate or moderate the relationship between internal work locus of control and bricolage.

## Conclusion

In summary, this study, albeit cross-sectional, does indeed show that social entrepreneurs’ internal work locus of control and bricolage should be vital triggers for the development of social entrepreneurship orientation. We conclude that bricolage is the alleyway through which internal work locus of control contributes to social entrepreneurship orientation. These findings address the gap in the literature on the determinants of social entrepreneurship orientation. Likewise, the findings do provide the opportunity for practitioners to develop exercises that facilitate bricolage to benefit social entrepreneurship orientation. The current study pinpoints limitations for future research.

## Data Availability Statement

The raw data supporting the conclusions of this article will be made available by the authors, without undue reservation.

## Ethics Statement

Written informed consent was obtained from the individual(s) for the publication of any potentially identifiable images or data included in this article.

## Author Contributions

PX and EH contributed to the conception and design of the study. RG organized the database and data collection. XS conducted the statistical analysis. EH wrote the first draft and handled final corrections. RG and XS wrote the methodology section under the guidance of PX. All authors contributed to manuscript revision, read, and approved the submitted version.

## Conflict of Interest

The authors declare that the research was conducted in the absence of any commercial or financial relationships that could be construed as a potential conflict of interest.

## Publisher’s Note

All claims expressed in this article are solely those of the authors and do not necessarily represent those of their affiliated organizations, or those of the publisher, the editors and the reviewers. Any product that may be evaluated in this article, or claim that may be made by its manufacturer, is not guaranteed or endorsed by the publisher.
